# Supplementary material for: Monitoring chronic inflammatory musculoskeletal diseases mixing virtual and face-to-face assessments—Results of the digireuma study
Source: PLOS Digit Health. 2022 Dec 7;1(12):e0000157. doi: 10.1371/journal.pdig.0000157 (PMC9931291; doi:10.1371/journal.pdig.0000157)
Supplement: S1 Text — (DOCX) [file pdig.0000157.s001.docx]

**S1 Text**. **Patients’ survey (focus group)**

a. Do you use Apps on your mobile phone or Tablet?

b. What types of Apps do you download?

c. Would you consider using an App to manage your rheumatoid arthritis? Please write down 2-3 things that you think would be most useful in such an App.

d. What would be useful to measure in such an App?

e. List the following choices from 1-10, in order of highest to lowest preference. Highest preference would be 1 and lowest would be 10.

i. What features would be useful in the application?

1. Appointment reminder.

2. Medication reminder.

3. Physical activity reminder.

4. Calculation of when to take blood tests prior to scheduled appointments.

5. Appointment manager.

6. The ability to send an e-mail message to one's regular doctor through the App.

7. Measurement of disease activity. The ability to view the activity indexes at any time, and not only before the consultation.

8. Detailed information about the disease.

9. Information on associations and services.

10. Connection with the hospital

ii. In terms of functionality, what things are most important to you?

1. Ease of use.

2. A user interface with large icons.

3. Simple-to-use functions.

4. No registration required.

5. Alarms.

6. Personalized.

7. Patient-hospital connection (see analytics).

8. The ability to connect or communicate with the physician.

9. That there is no need for the patient to manually complete any data requirements.

10. That the interface is very visual in nature.
