# Supplementary figures and images for: Monitoring chronic inflammatory musculoskeletal diseases mixing virtual and face-to-face assessments—Results of the digireuma study
Source: PLOS Digit Health. 2022 Dec 7;1(12):e0000157. doi: 10.1371/journal.pdig.0000157 (PMC9931291; doi:10.1371/journal.pdig.0000157)

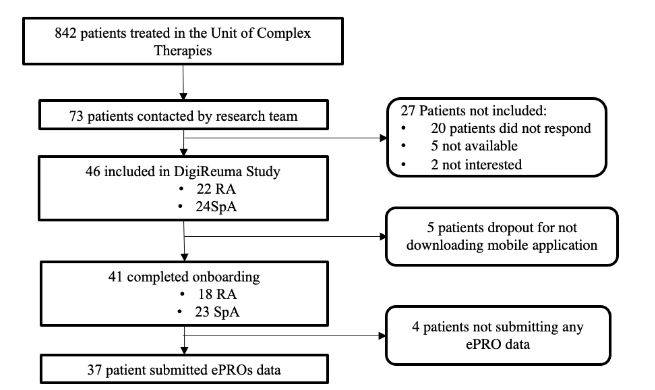

Supplement: S1 Fig — (PNG) [file pdig.0000157.s002.png]
