# Supplementary material for: Monitoring chronic inflammatory musculoskeletal diseases mixing virtual and face-to-face assessments—Results of the digireuma study
Source: PLOS Digit Health. 2022 Dec 7;1(12):e0000157. doi: 10.1371/journal.pdig.0000157 (PMC9931291; doi:10.1371/journal.pdig.0000157)
Supplement: S1 Table — (DOCX) [file pdig.0000157.s003.docx]

**S1 Table. Frequency of assessment of ePROs**

| Rheumatoid Arthritis | Spondyloarthritis |
| --- | --- |
| s-TJC: Every odd week on Mondays | s-TJC: Every odd week on Mondays |
| s-SJC: Every odd week on Mondays | s-SJC: Every odd week on Mondays |
| PtGA: Every odd week on Mondays | PtGA: Every odd week on Mondays |
| VAS pain: Every odd week on Tuesdays | VAS pain: Every odd week on Tuesdays |
| HAQ: Every even week on Mondays | BASDAI: Every odd week on Tuesdays |
| Physical activity (30 mins/ 3 times a week): Weekly on Sundays | ASAS HI: Every even week on Mondays |
| Past joint symptoms in last week: Monthly on Saturdays | Physical activity **(**30 min**s/** 3 times a week**)**: Weekly on Sundays |
| EMA anxiety: Weekly on Thursday and Thursday | Past joint symptoms in last week: Monthly on Saturdays |
| EMA depression: Weekly on Thursday and Thursday | EMA anxiety: Weekly on Thursday and Thursday |
| EMA fatigue: Weekly on Thursday and Thursday | EMA depression: Weekly on Thursday and Thursday |
| RA Flare: Always available | EMA fatigue: Weekly on Thursday and Thursday |
| Last 2 weeks treatment arthritis: Every odd week on Sundays | SpA Flare: Always available |
|  | Last 2 weeks treatment arthritis: Every odd week on Sundays |

SpA: spondyloarthritis; RA: rheumatoid arthritis; s-TJC: self-assessed Tender Joint Count; s-SJC: self-assessed Swollen Joint Count; PtGA: Patient Global Assessment of disease activity; VAS: Visual Analogue Scale; HAQ: Health Assessment Questionnaire; BASDAI: Bath Ankylosing Spondylitis Disease Activity Index; ASAS HI: Assessment of SpondyloArthritis International Society Health Index; EMA: Ecological Momentary Assesment
