# Supplementary material for: Monitoring chronic inflammatory musculoskeletal diseases mixing virtual and face-to-face assessments—Results of the digireuma study
Source: PLOS Digit Health. 2022 Dec 7;1(12):e0000157. doi: 10.1371/journal.pdig.0000157 (PMC9931291; doi:10.1371/journal.pdig.0000157)
Supplement: S2 Table — (DOCX) [file pdig.0000157.s004.docx]

**S2 Table. Clinical characteristics of patients in Digireuma as compared with standard of care**

|  | **RA patients** | | **SpA patients** | | **p-value*** | **p-value**** |
| --- | --- | --- | --- | --- | --- | --- |
|  | **Digireuma**  **(n=22)** | **Standard care**  **(n=241)** | **Digireuma**  **(n=24)** | **Standard care**  **(n=267)** |  |  |
| **Demographic and clinical features** |  | | | | | |
| **Sex (female)** | 18 (81.8) | 200 (83.0) | 10 (41.7) | 97 (36.6) | 0.8 | 0.6 |
| **Age (years)** | 41.9 (36.5, 48.2) | 52.8 (42.7, 60.3) | 35.1 (28.1, 39.9) | 44.4 (33.3, 54.4) | <0.01 | <0.01 |
| **Smoking habit (ever smoker)** | 12 (54.5) | 115 (47.7) | 9 (38.1) | 122 (45.7) | 0.8 | 0.6 |
| **RF positive** | 14 (63.6) | 188(87.0) |  |  | 0.01 |  |
| **ACPA positive** | 16 (72.7) | 182(84.2) |  |  | 0.3 |  |
| **HLA*B27 positive** |  |  | 13 (68.4) | 190 (71) |  | 0.7 |
| **Baseline measurements** |  | | | | | |
| **DAS28** | 4.5 (3.1, 5.3) | 5.2 (4.4, 6.1) |  |  | 0.01 |  |
| **HAQ** | 5 (1.8, 9.5) | 10.0 (6.0, 13.0) |  |  | <0.01 |  |
| **BASDAI** |  |  | 5.9 (5.4, 6.7) | 6 (5.0, 7.1) |  | 0.2 |
| **PhyGA** | 40.0 (27.5, 58.5) | 50.0 (30.0, 70.0) | 40.0 (30, 50.0) | 40.0 (20.0, 50.0) | 0.1 | 0.8 |
| **PtGA** | 50.0 (10.0, 56.0) | 52.0 (35.0, 70.0) | 70.0 (52.5, 80.0) | 70.0 (50.0, 63.3) | 0.1 | 0.8 |
| **CRP (mg/L)** | 4.3 (2.0, 7.9) | 6.2 (2.8, 16.6) | 5.0 (2.1, 19.0) | 4.7 (2.2, 13.0) | 0.01 | 0.7 |
