# Supplementary material for: Monitoring chronic inflammatory musculoskeletal diseases mixing virtual and face-to-face assessments—Results of the digireuma study
Source: PLOS Digit Health. 2022 Dec 7;1(12):e0000157. doi: 10.1371/journal.pdig.0000157 (PMC9931291; doi:10.1371/journal.pdig.0000157)
Supplement: Attachment — Submitted filename: Reply_reviewers_app29AUg2022_DEF.docx [file pdig.0000157.s005.docx]

**Reviewer #1:**

**Despite the feasibility nature of the present study, the paper of Benavent D et al. is the description of one of the most interesting real-life experiences of the use of digital health in the field of rheumatology in Europe.**

**It represents also a good example of application of telemedicine in the south of Europe, which is not obvious.**

**The data on a longer period of time will be interesting in order to understand the real benefit on the control of the diseases, such as the application of this technology in other diseases.**

**I think that this paper is suitable of publication after a minimal revision.**

**Despite the fact that the number of the final enrolled patients is small, the results are interesting and supports the use of mHealth for the cure of chronic arthritis.**

We thank the reviewer for such good feedback.

**Comment 1: Considering that the study is a good example of the application of remote care in clinical practice, I suggest to add somewhere a comment on the EULAR Ptc on telemedicine (https://nam12.safelinks.protection.outlook.com/?url=http%3A%2F%2Fdx.doi.org%2F10.1136%2Fannrheumdis-2022-222341&amp;data=05%7C01%7C%7C5c2d53c30f214ae07c3308da6e7f54c4%7C84df9e7fe9f640afb435aaaaaaaaaaaa%7C1%7C0%7C637943790405667936%7CUnknown%7CTWFpbGZsb3d8eyJWIjoiMC4wLjAwMDAiLCJQIjoiV2luMzIiLCJBTiI6Ik1haWwiLCJXVCI6Mn0%3D%7C3000%7C%7C%7C&amp;sdata=2endFUTmhQo9SnIkjG107QTayUJSwrZeeq4%2FYiAoBII%3D&amp;reserved=0).**

Thank you for your suggestion. The reference as well as some new content has been now added to the discussion, as follows:

“*More recently, the EULAR PtC for the development, prioritisation and implementation of telehealth for people with RMD have also been developed, identifying areas where telehealth may improve quality of care and increase healthcare access (15).* *These principles have been addressed throughout the development, evaluation, and implementation of the MAM and Adhera® for Rheumatology platform. This was achieved by first taking into account patients’ needs and requirements though the development of a focus group to address safety, relevance of the content and functionalities, and usability. Second, the involvement of healthcare professionals was made central to the project at the very outset, with the co-creation of a platform involving clinicians, researchers, digital health experts and technicians.”*

**-Patients**

**Comment 2: I would better clarify the clinical manifestations of enrolled patients with SpA which is a more heterogeneous disease than RA. Spondylitis or peripheral arthritis? Any extra-articular manifestations (also for RA)?**

Most patients included in the study had both axial and peripheral manifestations of the SpA. Extra-articular manifestations have been now included. Results have been adapted, as follows:

*“Among RA patients, 2/22 (10%) presented rheumatoid nodules, 1/22 (5 %) interstitial lung disease, 1/22 (5 %) Sjögren syndrome and 1/22 (5 %) autoimmune neutropenia. Out of the 23 patients with SpA, two were considered purely axial SpA, two purely peripheral SpA, and 19 have both axial and peripheral involvement. Concerning extraarticular manifestations in SpA, 4/24 (16.7 %) presented uveitis, 2/24 (8.3 %) psoriasis and 2/24 (8.3 %) inflammatory bowel disease.”*

**Comment 3: I have noticed that patients enrolled in the study had a milder disease in comparison with that of SoC group. Explain the reason which is not obvious.**

Thank you for this relevant comment. The previous comparison in Table 2 compared patients at the moment of app onboarding (Digireuma group) with patients at biologic start (SoC group). Hence, we previously acknowledged this as a limitation, as it read: “it is important to highlight that patient information for the latter was collected at baseline, which hinders any comparisons in regard to PROs.”

We have now established a comparison of patients in both group at the moment of biologic start, which can be seen in Supplementary Table S2 (the results have been adapted accordingly and the previous sentence has been removed from the discussion). As can be seen in this table, Digireuma and SoC groups had more similar outcome levels at biologic start. This holds true for all the parameter in the SpA group; in the RA group, there are still differences in some outcomes. Although patients were selected randomly from clinical practice to participate in Digireuma, we acknowledge that some of the more aged patients or with more comorbidity (including a worse disease control in general) may be less prone to participate in the study (which may bias these comparison). This potential bias was included as a limitation:

*“Patients in the Digireuma study presented some differences with those in the standard care population. Despite patients were selected randomly from clinical practice to participate in Digireuma, it is plausible that younger patients were more prone to participate in the study, with older patients declining participation, biasing the selection of patients.* *Further studies should look into the acceptance of the digital intervention across different demographics, especially those that might pose a health disparity. ”*

**Comment 4: No patients in b/tsDMARDs? Why? Was it a specific choice?**

This initial pilot study and the care protocol carried out (MAM protocol) is aimed at the Complex Therapies Unit of our Hospital. Thus, among the inclusion criteria, patients needed to have treatment with an advanced therapy (b/tsDMARDs) for at least one year. Hence, all patients were under b/tsDMARDs. In future phases of this project, it is planned to extend this approach to all patients with immune-mediated diseases, regardless of the type of treatment.

**-Physicians**

**Comment 5: A curiosity: the physicians who were available for the remote consultations were the same who followed up patients with f2f visits? Or there was a dedicated personnel for the digital part?**

In this project, two clinicians (rheumatologists) were actively involved. One of them was in charge of reviewing the incidents remotely. The other clinician carried out the telematic or face-to-face consultation

This has been added to the methods section:

“Two rheumatologists monitored these ePROs and patients were contacted for online or face-to-face interventions when deemed necessary by the clinicians (Figure 2). *Of note, one of them was in charge of reviewing the incidents remotely, while the other clinician carried out the telematic or face-to-face consultation*.”

**Comment 6: -I would do a comment about the % of participants which was low if we think about the total cohort that researchers had in mind. Do you think that some cultural barrier limited the participation?**

Thanks for the comment. We added this to the discussion:

“*Since this was the first stage of a pilot project, there were a few barriers that hampered the inclusion of more patients. Some of the contacted patients were reluctant to participate; 73 were contacted and 46 signed inform consent. In fact, a lower age of the patients enrolled in the study as compared to the standard care group indicates a potential reluctancy of older patients in the participation, indicating age as a barrier for the implementation of new technologies. In addition, another aspect that had a negative influence on recruitment was a potential fear of patients that their face-to-face visits will be replaced by telematic visits. COVID-19 pandemic might have been also a driver to adoption of the intervention; in one hand, some patients were more familiarized with technology; however, others might have been afraid that technology has been discussed as an alternative to face-to-face consultation. Hence, a potential cultural barrier on the use of these new technologies may have play a role in the number of patients participating in the study. Besides, human workforce who could devote time for this project was limited, both in the clinical and technical part. On top of this, clinicians have not received formal training on the implementation of these technologies, which made it more difficult to address the challenges along the project. Nonetheless, this study was not designed to gather insights on those aspects*.”

**Comment 7: I think that in this type of study, a comment on the socio-cultural environment has to be done in order to better understand the data. A comparison with other similar experiences but in other Countries can be interesting.**

We acknowledge the importance of the socio-cultural environment in the use of digital solutions, and would like to further explore into this issue in the future. The following has been added to the discussion:

**“***Thus, disease and socio-cultural factors may play an important role in the use of digital technologies; younger patients, with a long-standing diagnosis, and with stable disease activity might be more prone to use digital solutions. In this sense, there is a need to explore the quantification of engagement in digital interventions. Social determinants of health are undoubtedbly in this future research agenda. Hence, age should be considered since some RMDs may present in older patients. Perceptions and resistances on the use of telemedicine should be explored. Geography is a critical factor; although in our environment hospitals are accessible, many patients in some other areas may have mobility restrictions so there is a greater need for telemedicine implementation. One of the strengths of this study is that there was a meeting with a focus group of patients to identify whether the population was interested in digital tools and what unmet needs stood out in the follow-up. The mean age of the patients in the focus group was higher than that of the patients in the pilot study, which can be explained by one patient with outlier older age in the focus group which affected the mean, together with a general trend of older participants in the group due to logistics; as the meeting was face-to-face, the availability to attend in younger people due to job constraints was higher. In this sense, patients with SpA were not included in the assessment of the focus group. Patient profiles may also have had an impact on the pilot study. Due to the intrinsic characteristics of the disease, patients with SpA involved had a younger profile, with greater proportion of men, as compared with these with RA, which may have influenced their engagement in the solution. Future research should further investigate how social determinants of health impact the use of digital solutions in rheumatology. Besides, we would like to explore the quantification of engagement in digital interventions that include both an element of ePROs/monitoring and patient empowerment/education. In such case, we can foresee that engagement can also lead to lower symptom burden and potentially low interest in reporting ePROs on symptoms..”*

Concerning some other experiences, despite the growing interest in digital solutions, not many projects have developed in the field. We have added the following:

“*One of the most robust projects to date is the REmote MOnitoring of RA (REMORA) study, that aimed to test the feasibility of collecting daily patient-reported symptoms from 20 RA patients over 85 days using an app, being integrated in the electronic health record.(19) Seppen et al developed a twelve month, randomized clinical trial in RA patients to assess non-inferiority in terms of ΔDAS28 after 12 months and the ratio of the mean number of consultations with rheumatologists as compared to usual care patients. The study included 103 patients with stable low disease activity, and it showed that, in terms of DAS28, patient care backed by smartphone self-monitoring was comparable to standard care and reduced rheumatologist visits by 38%.(20*)”

19. Austin L, Sharp CA, van der Veer SN et al.Providing‘the bigger picture’: benefits and feasibility of integrating remote monitoring from smartphones into the electronic health record. Rheumatology (Oxford) 2019;59:367–78

20. Seppen BF, Wiegel J, Ter Wee MM, Schaardenburg D, Roorda LD, Nurmohamed MT, Boers M, Bos WH. Smartphone-Assisted Patient-Initiated Care Versus Usual care in Patients With Rheumatoid Arthritis and Low Disease Activity: a Randomized Controlled Trial. Arthritis Rheumatol. 2022 Jul 11. doi: 10.1002/art.42292. Epub ahead of print. PMID: 35818342.

**-App**

**Comment 8: Is it thinkable to use this app in a large scale?**

This was the first stage of the project, established as a feasibility study. As next steps, a 12-month follow-up multicentric study involving three hospitals is planned. This study will be complemented with a new project, a state funding project (PI22.00725) to carry out a multicenter project using this digital solution and based on the care protocol that we have designed (MAM protocol). This study will begin in January 2023, in which it is estimated to include a total of 240 patients and with an average follow-up per patient of 12 months.

**Comment 9: A comment on the costs of this feasibility study can be appreciated.**

This feasibility study did not include in its design a health economic assessment, which should have included cost related to healthcare delivery, digital solution, training, patient expenses (savings in transport), etc. The confidentiality of the contracts between the involved stakeholders do not allow to disclose the costs. As specified in the acknowledgments of the article, this pilot project could be carried out with an unrestricted grant from Abbvie mediated by the Spanish Society of Rheumatology. Of course, we find it very interesting in the future to assess whether a project with these characteristics, in addition to being feasible, is economically sustainable in the short and long term compared to standard clinical practice.

**Comment 10: Was this app used in the past for other health applications? If yes, spend few words on this.**

Thank you for this comment. Although the version of Adhera for Rheumatology was specifically designed for Digireuma, the background solution had indeed been used before. We added this sentence (methods, section Phase III):

“*In this study, we adapted the Adhera® for Rheumatology digital program, which was powered by the Adhera Digital Precision Digital Companion PlatformTM. The platform contains modules for personalized education, behavioral change and mental wellbeing that have been already used across multiple studies and implementations across different therapeutic areas (10-12). The adaptation of the digital intervention program for this study by a multidisciplinary team included rheumatologists, a general physician, psychologists, and digital health specialists.* ”

**Comment 11:**

**-Figure 2. I am not sure it is useful... it is not clear enough.**

Thank you for your suggestion. We have now adapted this figure to make it clearer.

**Reviewer #2: PLOS review**

**Monitoring Chronic Inflammatory Musculoskeletal Diseases Mixing Virtual and Face-to-Face Assessments - Results of the Digireuma Study**

**This study describes the development and pilot study of a Mixed Attention Model for remotely monitoring rheumatoid arthritis and spondyloarthritis. This is an important topic due to the large number of people affected and the intensive follow-up necessary to best handle these conditions.**

We thank the reviewer for his/her review.

**Abstract:**

**Comment 1:**

**The word “anytime” in the second to last sentence in the “Methods” subsection should be “any time.”**

Thank you for your comment, this has now been corrected.

**Comment 2:**

**In the “Results” subsection, please report the NPS rather than an overall rating with stars. The NPS is a standardized measurement tool that can be compared with other studies. There is nothing in the methods section that describes what question was asked of participants that would result in a star rating.**

The NPS has been now calculated and added, as well as an explanation of the star-rating in the methods section.

It has now been added to the abstracts and manuscript, section results:

*Abstract: “Regarding patient satisfaction, 65% of the respondents were considered to have endorsed Adhera® for Rheumatology, yielding a NPS of 57 and an overall rating was 4.3 out of 5 stars.”*

*Manuscript: “According to the NPS, 9/14 were considered promoters, 4/14 passives and 1/14 was a detractor, resulting in a NPS score of 57.”*

**Comment 3:**

**In the “Conclusion” subsection, consider changing the first sentence to read “****The use of the digital health solution is feasible in clinical practice to monitor ePROs for RA and SpA.”**

Thank you for your suggestion, the manuscript has been amended as proposed.

The sentence has been adapted in the abstract:

*“The use of the digital health solution is feasible in clinical practice to monitor ePROs for RA and SpA.”*

**Background:**

**Comment 4:**

**Rather than saying “thousands of people worldwide” please provide a more accurate estimated number. I would guess we are talking about hundreds of thousands.**

Thank you for your comment, we have now adapted the manuscript as follows:

*“Hundreds of thousands to millions of people worldwide are affected by such RMDs as rheumatoid arthritis (RA) and spondyloarthritis (SpA), with global prevalence estimates of 0.46% (95% confidence interval [CI] 0.39-0.54) and 0.2%-1.6%, respectively”.*

**Comment 5:**

**In the final sentence of the first paragraph, do you mean “mHealth” rather than “m-pHealth”? It is not clear what the latter term means.**

Indeed, we have now corrected the typo. Thank you.

**Comment 6:**

**In the final sentence of this section, please be consistent with the statistical analysis paragraph, which mentions a “feasibility” study, rather than a “proof-of-concept” study.**

The final sentence of this introductory section has been amended as proposed.

The sentence now reads:

*“The objective of this feasibility study was to test the use of ePROs to monitor disease activity, functionality, overall health, flares and problems with the medication as well as to evaluate user experience of patients with RA and SpA with this digital health solution."*

**Methods:**

**Comment 7:**

**Please consider reorganizing this section. The development of the protocol and the application seem to be mixed with the pilot test and the evaluation of the pilot test. I would suggest the following:**

**1. Development of protocol and application (focus group, Mixed Attention Model). It wasn’t clear whether the semi-structured interviews were part of the development process, or were done after the feasibility study.**

**2. Pilot test of the application (Digireuma Prospective Study)**

**3. Evaluation of the pilot test (Statistical analysis)**

Thank you for your comment. The structure of the methods section is currently based on the timeline of the project. In this sense, the sections follow your suggestion: Focus group, Mixed Attention Model, Digireuma Prospective Study, Statistical analysis. When we referred to “pilot” test, we meant the actual Digireuma Prospective Study- hence we adapted the terminology. Besides, there were two types of interviews, which may have led to confusion. We explain this further in comment 10.

**Comment 8:**

**The heading “Identification of unmet needs…” should probably be in italics.**

*Thank you, it has now been corrected.*

**Comment 9:**

**The methodology for the focus group needs to be better explained. How were people selected for the focus group? Who developed the focus group guide? Who facilitated the group? Was the discussion in the focus group transcribed and analyzed for themes?**

The methodology of the focus group is now further explained. Some of the sentences have been modified, as follows:

*“To establish the focus group, 20 patients with RA with a recent clinical visit were contacted by telephone* *in September 2019. The purpose of the meeting was explained to them and only five patients were available to attend a face-to-face meeting that lasted 120 minutes. (…)* *Two clinicians (DB, CP) developed the focus group guidelines and led the process. The clinicians facilitated the group with the aim to explore the unmet needs of patients, as well as their use of technology and preferences regarding mHealth.* *(…) The focus group meeting was held at HULP. Responses of the participants were transcribed and analyzed. The* *themes for the analysis of the transcriptions aimed to identify key patient needs and help define the set of ePROs to be incorporated into a digital solution. (…)”*

**Comment 10:**

**When were the semi-structured interviews completed? Were they done before or after the prospective study? The paper mentions the results of semi-structured interviews that were completed with participants following the study. Are these the same ones? If there are two different instances of semi-structured interviews, please describe how recruitment was done for the interviews.**

Two rounds of interviews were completed. First, in the development of the protocol and application, clinicians explored the habit of using digital solution and unmet needs of patients, through interviews to the focus group of 5 patients that were randomly recruited (reported in Focus group section). Secondly, as part of the assessment of the solution (in Digireuma Prospective Study section), semi-structured telephone interviews were conducted, in the middle of the total follow up (i.e. 3 months), to assess the feasibility and clinical relevance of the process in all recruited patients (thus, all patients were contacted). Some minor details have been adapted to facilitate the understanding throughout the manuscript.

Methods: “*After the 3 months follow-up, a semi-structured telephone interview was conducted to evaluate different items of the study.”*

*Results: “All 46 patients were contacted by telephone to make a semi-structured interview at 3 months, aiming to obtain feedback from patients and identify incidences with the digital solutions that could be improved by the research team. Only in one patient was not possible to contact despite several attempts. Eight patients (4 RA and 4 SpA) had technical problems because the automatic password recovery process failed, then they could not use the digital solution. In general, the remaining contacted patients (35 in total) expressed satisfaction with the initiative of the project and with the digital solution. The negative aspects most frequently reported were motivational messages (repetitive or not suitable) and reminders of PROs (the lack of feedback after completing them when disease is stable). The recurrent positive comments were having the possibility to inform the professional about incidents/evolution between clinical visits and having a source with reliable information about their disease. Among the suggestions for future improvements, the benefit of adding a calendar that included patient-tailored information and a more direct messaging tool for contacting healthcare professionals (not only for reporting flares) were highlighted.”*

**Comment 11:**

**Please provide examples of the motivational messages mentioned in the “MAM Implementation-Adaptation…” section. How was the input from patients obtained for the agreed-on set of ePROs?**

Thank you for the comment. The input from patients was obtained from the focus group, which explored the main domains that were interesting for patients. Future research should perform a deep qualitative analysis to look into a patient-centered design of the ePRO strategy to foster acceptability and compliance. The focus group meeting also supported the creation to a set of motivational messages addressing behavioral and self-management aspects.

We have added the following sentence including an example of one of the messages*: “These motivational messages addressed behavioral and self-management aspects, such as goal setting for physical activity. As an example of these: «How are you? To have a clear objective can help you to be more active. Set up an objective for exercising including how long or how many steps, when and the intensity you will do it. For example, “I will do brisk walking Tuesdays and Thursdays 30 minutes”* ».

**Comment 12:**

**In the section titled “Digireuma Prospective Study” please add the timeframe for the recruitment of patients. In the paragraph on Follow-up, please indicate how long patients were asked to use Adhera. It is not until the discussion section that I saw that there was a three-month period of use. In the final sentence of this section, you mention semi-structured telephone interviews, but not when they were completed and by whom. Were the interviews transcribed? Were they analyzed for themes?**

Thank you for your comment. Patients were recruited during one month, and for this initial phase were followed during three months (patients were asked to use the solution for at least six months). The main aim of the interviews at the end of the follow-up period was to assess patients’ satisfaction and any relevant issues on the use of the mobile solution.

The method section has been amended, as follows:

*(…)In order to evaluate the impact, use, and feasibility of Adhera® for Rheumatology in routine care, patients were consecutively recruited during a period of one month(…)*

*Patients were invited to participate for a maximum of 6 months of follow-up, but for this initial feasibility study, data were analyzed 3 months after recruitment. (…)*

*(…) Moreover, the feasibility and clinical relevance of the process was assessed at the end of the three-month follow-up by semi-structured telephone interviews conducted by the clinicians (DB, ChPR) to all recruited patients. These aimed to assess patients’ satisfaction as well as any relevant issues on the use of the mobile solution. (…)*

**Results:**

**Comment 13:**

**This section suffers from the same confusion between the focus group and the qualitative interviews as was in the methods section. The results section on the focus group refers to Table 1, which is titled to specify that these features were requested in qualitative interviews. Focus groups and qualitative interviews are not the same thing, and the paper should be consistent in referring to them.**

Thank you for this comment. We have adapted the title of Table 1 and the paper to be consistent with the term of focus group.

**Comment 14:**

**At the end of the section titled “Pilot-Testing Study,” you mentioned that patients in the Digireuma group were younger than those in the general cohort twice.**

The explanation referred to the stratified patients in RA and SpA groups. We have now created a Supplementary table for the comparison of patients in Digireuma and general cohort, and have adapted this paragraph, as follows:

*“In the RA group, there were no differences between patients in Digireuma and the general cohort for certain characteristics, such as gender, smoking habit, physician global assessment (PhyGA) or patient global assessment (PtGA). Nevertheless, the two groups differed in some relevant characteristics. For example, those in Digireuma were younger, presented worse DAS-28, C-reactive protein (CRP) and Health Assessment Questionnaire (HAQ) results. In the SpA group, there were no differences between groups in terms of gender, HLA*B27 positivity, or any of the assessed outcomes, including BASDAI, PhyGA, PtGA and CRP. However, patients included with SpA in Digireuma were younger.”*

**Comment 15:**

**The NPS is computed by subtracting the percentage of detractors from the percentage of promoters, 64% -7% = NPS of 57. It is not generally reported as fractions. What item was used for the general star rating of the app? This is never described in the methods section.**

Thank you for the comment. Concerning NPS, we added the following:

*“According to the NPS, 9/14 were considered promoters, 4/14 passives and 1/14 was a detractor, resulting in a NPS score of 57”.*

Concerning the general star rating, we added this sentence “The subjective quality of the application was measured using the MARS item. In particular, we asked “What is your general evaluation of the application?” which included a 5-start rating (one being the worse and 5 the best).”

We have added this to the methods:

*“The subjective quality of the application was measured using the Mobile Application Rating Scale (MARS) item (13). In particular, we asked “What is your general evaluation of the application?” which included a 5-start rating (one being the worse and 5 the best).”*

REF: https://mhealth.jmir.org/2015/1/e27

**Discussion:**

**Comment 16:**

**Why was the average age so much lower in the pilot test group than in the focus group?**

The focus group was composed of a small group of patients who had recently been seen at the CTU, of which finally only 5 were able to attend. This difference in age may be due to i) more difficulty to attend to the focus group meeting for younger people due to work activity or personal logistical difficulties (childcare...), ii) A patient with an outlier older age (78 years old) among the 5 focus group patient, who biased the mean of this group towards an old age. Of note, this patient was very engaged with digital technologies. We include this in the discussion:

*“**One of the strengths of this study is that there was a meeting with a focus group of patients to identify whether the population was interested in digital tools and what unmet needs stood out in the follow-up. The mean age of the patients in the focus group was higher than that of the patients in the pilot study, which can be explained by one patient with outlier older age in the focus group which affected the mean, together with a general trend of older participants in the group due to logistics; as the meeting was face-to-face, the availability to attend in younger people due to job constraints was higher.”*

**Comment 17:**

**At the end of the first paragraph in this section, you mention 4 out of 5 patients completing data using the digital solution, but Table 3 indicates a range of 69 to 88.9% for completion of ePROs. You state that “most” of the interventions were remote. Please provide a percentage or the actual number. You state that the majority of patients were considered promoters, but you should mention that only 14 patients rated the app using the NPS.**

Thank you for your comment. The paragraph has been amended as follows:

*(…) During the prospective 3-month follow up, 90% patients completed some data using the digital solution, with more than 7,000 interactions, of which 26 lead to an intervention (18 of them [70%] remote). Concerning usability and satisfaction, although only 14 patients rated the solution, the majority of them (9 patients [64%]) were considered promoters.*

**Comment 18:**

**Please type out the source of the acronym “EULAR PtC.”**

It is specified in the background section, in the first apparition of the acronym:

*In this regard, the European Alliance of Associations for Rheumatology (EULAR) published the EULAR points-to-consider (PtC) guidelines for the development, evaluation and implementation of apps aiding self-management of RMDs(8).*

**Comment 19:**

**In the second to last paragraph, you mention NPW. Did you mean NPS?**

Indeed, we referred to NPS. It has now been corrected.

**Reviewer #3: Benavent et al. reported the performance of a hybrid (virtual and face-to-face) monitoring strategy for personalized care in rheumatoid arthritis (RA) and spondyloarthritis (SpA), which has implemented a mobile app to control the patient reported outcomes in pateints with SpA and RA. The paper is important given the general trend for the digitalization in healthcare and scarcity of data on digital solutions in rheumatology. Nevertheless, there are several points that have to be addressed by authors.**

We thank you for your in-depth review and for your valuable comments.

**Comment 1:**

**Authors claim „Thousands of people worldwide are affected by such RMDs as rheumatoid arthritis (RA) and spondyloarthritis (SpA)”. This is an underestimation, given the estimated global prevalence of RA of around 0.5% (Almutairi K, Nossent J, Preen D, Keen H, Inderjeeth C. The global prevalence of rheumatoid arthritis: a meta-analysis based on a systematic review. Rheumatol Int. 2021;41(5):863-877) and of SpA ranging between** **0.2% to 1.6% (Stolwijk C, van Onna M, Boonen A, van Tubergen A. Global Prevalence of Spondyloarthritis: A Systematic Review and Meta-Regression Analysis. Arthritis Care Res (Hoboken). 2016;68(9):1320-1331). So, the phrase may give a reader a wrong impression, as there are hundreds of thousands to millions of people affected with the diseases, not thousands.**

Thank you very much for your comment and suggestions. The sentence has been amended as follows:

*Hundreds of thousands to millions of people worldwide are affected by such RMDs as rheumatoid arthritis (RA) and spondyloarthritis (SpA), with global prevalence estimates of 0.46% (95% confidence interval [CI] 0.39-0.54) and 0.2%-1.6%, respectively.*

**Comment 2:**

**Authors claim that 2 clinicians (DB, CP) were involved in the focus group. I am struggling to identify who is referred to as CP, as there is no co-author listed with such initials.**

CP stands for the last author, Chamaida Plasencia; since her full name is Chamaida Plasencia Rodríguez, we have adapted the acronym as ChPR for better clarification.

**Comment 3:**

**Authors claim that 5 patients were involved in the focus group. Could the authors please clarify how the size of the focus group was determined to make sure it depicts the real-life picture, and how the participants were selected?**

Thank you for your comment. The objective of the focus group was to g assess potential benefits of using digital solutions for patients with RMDs and unmet needs in this field. We did not calculate a predetermined size since the literature on determining sample size for a focus group inconsistent; we contacted 20 patients by telephone who have recently been visited. As the meeting was face-to-face (it was pre-COVID) only 5 were able to attend.

We further explain this in the methods:

*“To establish the focus group, 20 patients with RA with a recent clinical visit were contacted by telephone* *in September 2019. The purpose of the meeting was explained to them, and only five patients were available to attend a face-to-face meeting that lasted 120 minutes. Patients with different ages were involved in this focus group. Only patients with RA were included because this pilot project was initially addressed to this disease.”*

**Comment 4:**

**In the Results section, authors report that “The focus group included 5 patients with RA”. In the methods section, authors report that altogether 5 patients were included in the focus group. Does this mean that no patients with SpA were included in the focus group, so the SpA patients sis not participate in the selection of PROs and the frequency of their assessment for SpA (unlike RA)? If so, could the “Identification of unmet needs- Focus group” section be corrected accordingly?**

The focus group was initially done only in RA because at the beginning of the project, when this meeting was held, the solution was only intended to be applied in this disease. However, when the protocol of the clinical project was developed, it was decided to carry it out in the two most frequent diseases in our unit, namely RA and SpA. Regarding PROs, in the case of patients with RA the most used in clinical practice were considered and discussed with patients. In the case of SpA, those recommended by scientific societies and those most used in clinical practice were included.

We have adapted the methods accordingly, and have included this in the discussion:

Methods: “*Healthcare professionals from the CTU had raised these concerns and leading to the formation of a focus group of patients with RA.”(…) “Later, in the study design, patients with SpA were also included because both are the most frequent pathologies under b/tsDMARDs in our unit.”*

Discussion: “*Patients with SpA were not included in the assessment of the focus group*.”

**Comment 5:**

**In the Sub-Chapter “Conceptualization of a Mixed Attention Model”, authors provide the reference as ISBN:978-84-09-38502-7. Unfortunately, I am struggling to find the source they are referring to both using search engines and ISBN identificators. Could author please cite the book they are referring to in a more convenient way or provide the description of face-to-face visits and virtual follow-ups in detail in the text?**

The protocol was registered in the *Agencia del ISBN en España*, and it can be accessed online in this link:

<https://www.culturaydeporte.gob.es/webISBN/tituloDetalle.do?sidTitul=2784906&action=busquedaInicial&noValidating=true&POS=0&MAX=50&TOTAL=0&layout=busquedaisbn&language=es&prev_layout=busquedaisbn>), although not read in its English version. Besides, we include a translated version of the protocol for your review.

As it can be read in the protocol:

“Patients are provided with access to a digital solution that can be downloaded free of charge and whose design has been developed and supervised by the professionals involved in the CTU. Then, patients can complete the questionnaires of activity, disability, and other domains as patient-reported outcomes (PRO) and get reliable information about their pathology and functional capacity, as well as other interesting aspects impacting their daily lives.

The incidents recorded in the tool are periodically reviewed on a web interface by a clinician whose clinical work is partly dedicated to this purpose (digital clinician). This professional, or another professional from the CTU (on-site clinician), contacts the patient by telephone to resolve the reported incident. The incident may be resolved completely remotely, or a face-to-face visit may be arranged to address the problem.”

**Comment 6:**

**From the Supplementary Table 1, it is clear that different** **patient-reported outcomes were not accessed at once but on different days. Could the authors please provide the reason for such assessment and mention the problems related to it in the discussion (e.g. problems with potential use of data to calculate disease activity indices such as ASDAS, as it supposes that PROs included in the index – BASDAI and PtGA – are registered at the same moment of time)?**

Thank you for this comment. With the objective of not overwhelm patients with a high burden of questionnaires, patient-reported outcomes were displayed with the timing shown in Supplementary Table 1. We acknowledge the pitfalls for the calculations of disease activity indices, such as DAS28 or ASDAS. We have now commented this on the discussion:

*“Besides, different patient-reported outcomes were displayed periodically and not accessed at once by patients, which hampers the calculations of disease activity indices, such as DAS28 or ASDAS.”*

**Comment 7:**

**In the Methods section, authors write that Bath Ankylosing Spondylitis Functional Index (BASFI) was administered to1 the patients. At the same time, BASFI is not included in the Supplementary Table 1. Could the authors please address this discrepancy?**

Initially, BASFI was intended to be included among the questionnaires, but finally it was decided not to be included to limit the burden of response of participants. However, for next stages, we consider it to be relevant. We have amended the manuscript by deleting BASFI assessment from methodology.

**Comment 8:**

**Authors claim: “The characteristics of the patients in the Digireuma study were compared to the baseline characteristics of a sample cohort from the UCT, matched by disease.” Could the authors please specify what cohort are they referring to and characterize it, as the abbreviation “UCT” is not explained in the text and no reference provided?**

The acronym UCT referred to the previously mentioned “Complex Therapy Unit”, but included a typo since it should read CTU. It has now been corrected. The Complex Therapy Unit attends patients with RMDs treated with b/tsDMARDs. Among them, there are patients who have RA and patients who have SpA. Patients belonging to the CTU followed by standard of care were included in these groups.

We have now compared data at baseline of biologic initiation in both groups. These results can now be seen in Supplementary Table S2.

**Comment 9:**

**In the result section, authors report that the mean age of the included patients (48± 12 and 42± 9 years in the RA and SpA groups) was significantly different from the mean age of the focus group (62 ± 16.7 years). Could the authors please comment on such a discrepancy and whether they see it as an important one?**

Thank you for this comment. Of note, a group of patients who had recently been seen at the CTU were selected for the focus group; although 20 were contacted, finally only 5 were able to attend. This difference in age may be due to i) more difficulty for younger patients to attend to the focus group meeting due to work activity or personal logistical difficulties, ii) A patient with an outlier older age (78 years old) among the 5 focus group patient biased the mean of this group towards an old age. Of note, this patient was very engaged with digital technologies. We include this in the discussion:

*“One of the strengths of this study is that there was a meeting with a focus group of patients to identify whether the population was interested in digital tools and what unmet needs stood out in the follow-up. The mean age of the patients in the focus group was higher than that of the patients in the pilot study, which can be explained by one patient with outlier older age in the focus group which affected the mean, together with a general trend of older participants in the group due to logistics; as the meeting was face-to-face, the availability to attend in younger people due to job constraints was higher”.*

**Comment 10:**

**Authors report that there was a significant difference in the number of interactions between RA and SpA groups, especially in the number of completed questionnaires (RA - 2,178 questionnaire items, SpA - 1637 questionnaire items; patients with RA completed a median of 9.5 (4.3, 15.8) ePROs during the 3-months, while patients with SpA completed a median of 3 ePROs (1, 12)), although the frequency of the administered questionnaires was the same between the groups (every second week), and SpA patients had more PROs to fill in (so more answers expected). Could the authors please explain such a difference between the patient groups?**

Thank you for your comment. The percentage of patients with at least one entry was similar in both groups, close to 90% in both cases. However, patients with SpA responded less frequently to questionnaires. Although we do not have a clear explanation, we hypothesized that i) disease activity in both groups yielded differences in attrition; although not directly comparable, it seems that the RA group might have worse baselines outcomes, and this may have affected the profile of patients, ii) type of questionnaire: PROs in the SpA group were longer to complete (BASDAI and ASAS-HI as opposed to pain VAS and HAQ), which may have contributed to less motivation in the SpA group to fulfil questionnaires. iii) Baseline characteristics of patients with RA and SpA were different; patients with SpA had a younger profile, with greater proportion of men. Besides, patients with SpA had not been included in the focus group, and this may have led to a less adequate selection of relevant outcomes for patients. We have now included this in the discussion:

“*In this sense, patients with SpA were not included in the assessment of the focus group. Patient profiles may also have had an impact on the pilot study. Patients with SpA involved had a younger profile, with greater proportion of men, as compared with these with RA, which may have influenced their engagement in the solution.”*

**Comment 11:**

**As authors explore feasibility of the proposed digital assessment, I would be very much interested in the “retention rate”, which authors unfortunately report only partially (of 46 patients, 5 did not download the application, and 4 did not submit at least one entry). Could the authors please provide the number of patients who completed all the prescribed PROs, as well as the number of patients who have completed at least 75% and at least 50% of the prescribed PROs?**

Thank you for the comment. We would like to be cautious about considering ePROs reporting as retention rate of this digital solution/intervention which is not only intended to monitor but to empower patients. In fact, the response to ePROs was optional and the number of available ePRO was exploratory in the sense that we expected that not all participants would have reported all ePROs. In fact, none of the patients completed 100 % of the items, and a minority (4/46, 8.7 %) completed more than 75 % of the items.

We added this sentence to the discussion “Besides, we would like to explore the quantification of engagement in digital interventions that include both an element of ePROs/monitoring and patient empowerment/education. In such case, we can foresee that engagement can also lead to lower symptom burden and potentially low interest in reporting ePROs on symptoms*.”*

**Comment 12:**

**For the feasibility assessment, physician’s perspective is missing. Could authors please comment on the working load of “the digital clinician”: how time consuming for was it to check the data provided by patients and to contact patients?**

We have not calculated the exact time that each intervention took in real clinical practice. However, based on the weekly time that was allocated for this project, we estimate that the digital clinician has spent 1 hour per week reviewing incidents on the platform and the clinician has taken an average of 1 hour per week to resolve incidents telematically and/or face-to-face for this sample of 46 patients.

**Comment 13:**

**As can be seen in the Table 2, both patients with RA and SpA included in the study were having a significantly milder disease as compared to the standard care (e.g. DAS28 2.9 vs. 5.2 for RA patients, BASDAI 2.1 vs. 6 for SpA patients). Therefore, the possibility to generalize the findings to the general population of patients with RA and SpA, as well as the feasibility of the digital assessments (as more severe patients would report more flares) are questionable. Could authors please comment on that issue?**

Baseline analysis shown in former Table 2 compared drug start baseline of patients of SoC group against app baseline of Digireuma patients. Hence, as mentioned in the previous discussion, “, it is important to highlight that patient information for the latter was collected at baseline, which hinders any comparisons in regard to PROs.” However, we have adapted this material to make adequate comparisons, with both groups of patients at biologic start baseline, in Supplementary Table 2. The following paragraph has been adapted in the results:

“*In the RA group, there were no differences between patients in Digireuma and the general cohort for certain characteristics, such as gender, smoking habit, physician global assessment (PhyGA) or patient global assessment (PtGA). Nevertheless, the two groups differed in some relevant characteristics. For example, those in Digireuma were younger, presented worse DAS-28, C-reactive protein (CRP) and Health Assessment Questionaire (HAQ) results. In the SpA group, there were no differences between groups in terms of gender, HLA*B27 positivity, or any of the assessed outcomes, including BASDAI, PhyGA, PtGA and CRP. However, patients included with SpA in Digireuma were younger.”*

**Comment 14:**

**Although PROs are used widely to determine the disease activity of both SpA and RA, using PROs only for defining disease activity (and therefore treatment strategy) may have its own disadvantages. It is well known that most common manifestations of both RA and SpA (such as pain) may have other reasons apart from SpA and RA itself, such as concomitant fibromyalgia, osteoarthritis, depression, other conditions. A** **significant discrepancy between self-reported and physician-measured arthritis, especially for swollen joint count, was also previously demonstrated (Cheung PP, Gossec L, Mak A, March L. Reliability of joint count assessment in rheumatoid arthritis: a systematic literature review. Semin Arthritis Rheum. 2014;43(6):721-729). How did authors address this issue? Were patients instructed to differentiate between the manifestations of the rheumatic disease and other causes of musculoskeletal symptoms? Were comparisons performed between patient-reported and physician-detected joint problems?**

We thank you for your comment and agree with it. Assessing disease activity only with PROs may be certainly a limitation, and particularly with joint counts. Concerning PRO questionnaires, the outcomes used were the same as those used in daily clinical practice, so that patient subjectivity should be in principle similar in the face-to-face and digital assessment. Besides, all included patients had been in the CTU for at least 1 year and were previously trained to fill out the questionnaires.

This stage of Digireuma was only the first step towards a more ambitious goal in telemonitoring, in which we are following patients through more visits, and we plan to assess the reliability of patient reported results with physician outcomes. Although not in the objectives of this first-phase of this feasibility study, we acknowledge it can be understood as a shortcoming of the study, and have therefore included it as a limitation:

*“Furthermore, a significant discrepancy between self-reported and physician-measured arthritis has been reported in previous studies (23), which may limit the validity of the joint-count results.* *This might also indicate an opportunity for health literacy interventions to support the self-monitoring by patients.”*
